# Addressing the Limitations in Mobile Health Application Research for Oral Cancer Knowledge

**DOI:** 10.1002/hsr2.70516

**Published:** 2025-02-19

**Authors:** Carlos M. Ardila, Pradeep K. Yadalam

**Affiliations:** ^1^ Basic Sciences Department, Biomedical Stomatology Research Group, Faculty of Dentistry Universidad de Antioquia U de A Medellín Colombia; ^2^ Department of Periodontics, Saveetha Dental College and Hospital, Saveetha Institute of Medical and Technical Sciences (SIMATS) Saveetha University Chennai Tamil Nadu India

To the Editor,

We commend the authors of the article “The Types and Effectiveness of Mobile Health Applications Used in Improving Oral Cancer Knowledge: A Mixed Methods Systematic Review” for addressing a critical area in public health [1]. This study is a timely exploration of the potential of mobile health applications to enhance oral cancer (OC) knowledge. However, we would like to offer constructive feedback to improve the clarity and comprehensiveness of the reported findings.

The systematic review included three articles from two studies, all conducted in India, with literature published exclusively in English [2, 3, 4]. While this provides valuable insights into a specific demographic, the limited geographic scope constrains the global applicability of the findings. We suggest that the authors explicitly address this limitation in the discussion section and underscore the need for further research across diverse populations and settings.

Of the three included articles, only one utilized a qualitative design [2], while the remaining two employed quantitative nonrandomized approaches [3, 4]. This heterogeneity in study designs may have influenced the robustness of the synthesized findings. Furthermore, only one study explicitly reported the use of a prevalidated questionnaire, a crucial aspect for ensuring methodological rigor [4]. The implications of these methodological disparities on the review's conclusions warrant further critical discussion.

The review offers detailed technical and functional specifications for the two applications examined—M‐OncoED and Prayaas. However, the lack of clarity about the offline functionality of M‐OncoED and its compatibility with basic handsets raises concerns about accessibility, especially in low‐resource settings. Incorporating such information would enhance the understanding of these applications' feasibility for broader implementation.

The findings on the effectiveness of these applications are mixed. M‐OncoED demonstrated limited effectiveness in improving OC knowledge but showed positive behavioral outcomes, such as an increase in the provision of OC screening advice. Conversely, Prayaas significantly improved knowledge outcomes but lacked data on its clinical and epidemiological impact [1]. A more nuanced presentation of these findings could provide a balanced and comprehensive interpretation of the applications' effectiveness.

While the authors emphasize the promise of mobile health applications [1], the review's conclusion does not sufficiently highlight the need for robust randomized controlled trials with larger sample sizes and diverse populations. Including this recommendation would provide critical guidance for future studies aiming to expand the evidence base in this field.

In summary, while this review underscores the potential of mobile health applications to enhance OC knowledge, addressing the aforementioned concerns could further strengthen the reliability and applicability of its conclusions. We hope these observations contribute constructively to ongoing discussions about mobile health innovations in cancer prevention.

## Author Contributions

**Carlos M. Ardila:** conceptualization, investigation, funding acquisition, writing–original draft, methodology, validation, visualization, writing–review and editing, formal analysis, project administration, supervision. **Pradeep K. Yadalam:** conceptualization, investigation, funding acquisition, writing–original draft, methodology, validation, visualization, writing–review and editing, formal analysis, supervision.

## Ethics Statement

The authors have nothing to report.

## Conflicts of Interest

The authors declare no conflicts of interest.

## Transparency Statement

The lead/Corresponding author affirms that this manuscript is an honest, accurate, and transparent account of the study being reported; that no important aspects of the study have been omitted; and that any discrepancies from the study as planned (and, if relevant, registered) have been explained.

## Data Availability

The authors have nothing to report.
